# 10.C. Round table: Is public health just science?

**DOI:** 10.1093/eurpub/ckac129.630

**Published:** 2022-10-25

**Authors:** Organised by: UK Faculty of Public Health, EUPHA-ETH, EUPHA-PHPP, Chair persons: Farhang Tahzib (EUPHA-ETH)

## Abstract

Politicians and policy makers often assert that they are following the science and the evidence in their decision making. Public health is proud of its rigorous scientific and evidence-based approaches and providing the numbers and facts. It is clear though that such numbers and facts, as during the pandemic, have resulted in diverse policies in different countries. Some commentators have noted that public health has lost its way, arguing that its leadership is failing, lacks courage and not sufficiently concerned with issues about social reform and political accountability and change. Is providing facts and the science sufficient to fulfil our mandate and obligations in public health? What should be our role in advocacy and engagement in politics and policy-making to promote and protect the public's health and tackle health inequalities? The purpose of the roundtable is to explore questions of whether public health is “just science”: is it built merely on a scientific skillset; or is it about justice-promoting science? This question goes to the heart of public health's mandate, with direct implications for public health practice. The questions will be considered largely from the perspective and evidence from the Covid pandemic. There will be presentations by senior distinguished academics and public health practitioners and leaders who have been researching and advising policy makers on wide range of public health issues. They will share their research and experience in particular from membership of the UK Independent Scientific Advisory Group (Indie_ SAGE) for the pandemic and on the Pandemic Ethics Accelerator, as well as other groups advising the government and public organisations on the analysis and implementation of policy options during the pandemic. This will be followed by reflections and discussion with the workshop participants and their reflections and insights on these critical questions.

**Key messages:**

• Public health is not merely a technical, scientific discipline; it incorporates norms and values, and operates in a political environment where value-judgments are fundamental to decision making.

• Public health practitioners and leaders need to consider advocacy and engagement, and the moral foundations of public health, as key to their mandate and professional obligations.

**Speakers/Panellists:**

**John Coggon**

University of Bristol, Bristol, UK

**Martin McKee**

LSHTM, London, UK

**Marleen Bekker**

Wageningen University & Research, Wageningen, Netherlands

**Farhang Tahzib**

Faculty of Public Health, Haywards Heath, UK

